# Altered detrusor contractility in MPTP-treated common marmosets with bladder hyperreflexia

**DOI:** 10.1371/journal.pone.0175797

**Published:** 2017-05-17

**Authors:** Sara Pritchard, Michael J. Jackson, Atsuko Hikima, Lisa Lione, Christopher D. Benham, K. Ray Chaudhuri, Sarah Rose, Peter Jenner, Mahmoud M. Iravani

**Affiliations:** 1 Department of Pharmacy, Pharmacology and Postgraduate Medicine, University of Hertfordshire, Hatfield, United Kingdom; 2 Neurodegenerative Disease Research Group, Institute of Pharmaceutical Sciences, Faculty of Life Sciences and Medicine, King’s College London, London, United Kingdom; 3 King’s Health Partnership, London, United Kingdom; Florey Institute of Neuroscience and Mental Health, The University of Melbourne, AUSTRALIA

## Abstract

Bladder hyperreflexia is a common non-motor feature of Parkinson’s disease. We now report on the contractility of the isolated primate detrusor strips devoid of nerve input and show that following MPTP, the amplitude and frequency of spontaneous contraction was increased. These responses were unaffected by dopamine D_1_ and D_2_ receptor agonists A77636 and ropinirole respectively. Contractions by exogenous carbachol, histamine or ATP were similar and no differences in the magnitude of noradrenaline-induced relaxation were seen in detrusor strip obtained from normal and MPTP-treated common marmosets (*Callithrix jacchus*). However, the neurogenic contractions following electrical field stimulation of the intrinsic nerves (EFS) were markedly greater in strips obtained from MPTP treated animals. EFS evoked non-cholinergic contractions following atropine were also greater but the contribution of the cholinergic innervation as a proportion of the overall contraction was smaller in the detrusor strips of MPTP treated animals, suggesting a preferential enhancement of the non-cholinergic transmission. Although dopaminergic mechanism has been proposed to underlie bladder hyperreflexia in MPTP-treated animals with intact bladder, the present data indicates that the increased neurogenically mediated contractions where no extrinsic innervation exists might be due to long-term adaptive changes locally as a result of the loss of the nigrostriatal output.

## Introduction

Urinary dysfunction is common in Parkinson’s disease (PD) and takes the form of nocturia, frequency and urgency of micturition associated with detrusor hyperreflexia [1, 2]. Loss of dopaminergic nigro-striatal input is thought at least partially responsible for the hyperreflexia and imaging studies show changes in dopaminergic function in the striatum and in its output to the globus pallidus in individuals with PD and bladder dysfunction compared to those with PD and normal bladder function [3, 4]. In fact, the extent of striatal denervation as measured by dopamine transporter loss correlates with the severity of bladder dysfunction. More specifically, the D_1_ receptor mediated output from the striatum to the internal segment of the globus pallidus appears inhibitory on micturition while the D_2_ receptor mediated output to external globus pallidus and then to the subthalamic nucleus is facilitatory [5]. Much of the evidence for this has arisen from experimental studies in animals. Unilateral lesions of the nigro-striatal dopaminergic pathway using 6-hydroxydopamine in the rat cause bladder hyperreflexia as does the administration of the selective nigral dopaminergic neurone toxin, 1-methyl-4-phenyl-1,2,3,6-tetrahydropyridine (MPTP) to primate species [6–10]. Electrical stimulation of the substantia nigra and the direct injection of dopamine into the striatum can reverse these changes. In both man and in experimental models of PD, D_1_ dopamine agonists improve bladder hyperreflexia whereas D_2_ agonists either have no effect or make it worse. All of these data contribute to the idea that a centrally mediated dopaminergic mechanism underlies hyperreflexia in PD.

The evidence for basal ganglia involvement in hyperreflexia in PD suggests that bladder function should be rectified by treatment with L-dopa and dopamine agonist drugs. Bladder over-activity can be improved using dopamine replacement therapy in some individuals but not all. The effects are complex and perhaps biphasic with postulated roles for D_1_ and D_2_ receptors, auto-receptors and post-synaptic sites and both central and peripheral dopamine receptor stimulation [5, 7]. This presumably reflects the complexity of the control of bladder function at multiple levels. Indeed, the regulation of micturition is highly complex being dependent on the autonomic arc of the sacral spinal cord segments but tonically facilitated by the pontine micturition centre with the storage function facilitated by the hypothalamus, cerebellum, frontal cortex as well as the basal ganglia [11–13]. Perhaps surprisingly, there has been little research into changes in bladder control in PD in these other brain regions or in the periphery. One possibility is that long-term loss of striatal dopaminergic innervation either directly or indirectly leads to changes in the innervation and/or responsiveness of smooth muscle in bladder. But so far, no study has investigated whether changes occur in isolated detrusor muscle contractility in either man or in primate models of PD.

Similarly, the innervation and responsiveness of bladder smooth muscle to cholinergic and non-cholinergic neurotransmitters controlling its contractility have not been examined in PD. The neuronal input to the bladder is usually described as being mediated by parasympathetic cholinergic neurones together with sympathetic adrenergic and noradrenergic innervation [14]. This is reflected in the reliance on antimuscarinic drugs for the treatment of bladder dysfunction in PD [11–13]. But in reality, changes in other forms of innervation may be a greater contributor to hyperreflexia than the cholinergic system. For example, there may be alterations in the non-adrenergic, non-cholinergic neurotransmission, presumed to be purinergic [15], that reflects the atropine-resistant contraction of the detrusor found in rodents, primates and man [16–19].

For these reasons, we have investigated local changes in bladder function in a non-human primate model of PD, namely the MPTP-treated primate where bladder hyperreflexia occurs and where bladder enlargement is commonly observed [6]. We now report on changes in isolated bladder contractility occurring as result of striatal dopaminergic denervation. We have also performed an initial investigation of the changes occurring in neurotransmitter responsiveness of isolated detrusor muscle strips and alterations in EFS contractility as a measure of altered innervation. As these studies were carried out in primates only those major neurotransmitter systems involved in the control of bladder function could be investigated.

## Materials and methods

### Animals

Two groups of normal, control (n = 7; 3 male and 4 female, 333–407 g; 373±16 g) and 1-methyl-4-phenyl-1,2,3,6-tetrahydropyridine (MPTP; Sigma, Poole, UK)-treated (n = 7, 2 males and 5 female, 313–389 g; 359±17 g) (MPTP, 5 x 2mg/kg s.c.) adult common marmosets (*Callithrix jacchus*, Harlan, UK) were used in this study. The MPTP-treated animals were prepared according to previously published protocols [20, 21] and were used in other studies where the symptomatic effects of various dopamine agonists were examined. MPTP-lesioned animals were culled between 1 to 3 years after the end of MPTP treatment, so there was no likelihood of the presence of residual MPTP. The normal marmosets were completely drug and toxin naïve.

All marmosets were kept in home cages with dimensions of height: 166, width: 140 and depth: 90 cm at an ambient temperature of 25° ± 1° C. Animals were housed in home cages in pairs, as approved by the Home Office inspectorate at King’s College London facilities, in a 12h light/dark cycle at an ambient temperature of 25±1° C and were fed once daily with a diet of bananas, oranges and apples and had free access to food pellets (Mini Marex–E; Special diet Services) and drinking water.

The animals’ environment was enriched by installation of viewing turret on top of the cages to mimic height as would be the case in a normal habitat (height: 36cm width: 35cm depth: 50cm) and wooden ladders/perches, Hammocks, swings, nesting boxes, multiple feeding platforms and saw dusted floors for forage feeding.

All experimental work was carried out in accordance with the Animals (Scientific Procedures) Act 1986 approved by the Kings College London Ethical Review Committee. In particular the primate experiments reported were subject to and were carried out under the Animals (Scientific Procedures) Act 1986 under a Home Office Project Licence (PPL 70/4986) approved by the King’s Ethical Committee which complied fully with the guidelines and recommendations set out in the Weatherall Report 2006 –the use of non-human primates in research (https://royalsociety.org/policy/publications/2006/weatherall-report/).

### Organ bath studies

Common marmosets were killed using overdose of pentobarbital sodium (60 mg/kg; Euthatal, Merial Animal Health Ltd.) between 7:30 and 8:30 am. Upon cessation of foot and corneal reflexes, the thoracic and abdominal cavities were opened. The animals were transcardially perfused with ice-cold oxygenated (95% O_2_ plus 5% CO_2_) Krebs-Henseleit solution (composition mM: NaCl 118, KCI 4.7, CaCl_2_ 2.5, MgSO_4_ 1.2, NaHCO_3_ 25, KH_2_PO_4_ 1.2, glucose 11) and the bladder was excised whole above the level of ureter and placed in this solution. While still in aerated Krebs-Henseleit solution, three to four 1 cm lengths of detrusor were suspended in a 15 ml organ baths in parallel, at a resting tension of 1.0 g force at 37°C according to previously published reports [18, 22]. Following a 30 min equilibration period, the contractile activity of the detrusor strips was measured using an isometric transducer connected to LabChart data acquisition system (AD Instruments Ltd., Oxford, UK).

Spontaneous rhythmic activity of the detrusor was assessed by calculating the amplitude and frequency of spontaneous contractions. The amplitude was estimated by adding together the tension of individual contractions occurring during a period of 10 min and dividing this sum by the number of contractions during this period. The rate of spontaneous activity (rate/min) was derived from dividing the total number of contractions during the 10-min observation time.

To assess whether prior MPTP treatment affected the receptor/effector coupling or whether there were changes at the level of smooth muscle, the detrusor preparation was contracted either directly by cumulative addition of various concentrations of carbachol (0.01 to 30 μM; Sigma Aldrich) or adenosine triphosphate (ATP; 1 mM; Sigma Aldrich). The bladder also receives an adrenergic input, which upon release of noradrenaline causes the relaxation of the detrusor [23]. Therefore, to investigate whether there are changes in the responsiveness of the detrusor preparations from the normal and MPTP-treated animals, we examined the effects of noradrenaline, 10μM on the basal tone of the detrusor strips. In order to test contractility independently of the cholinergic or non-adrenergic, noncholinergic (NANC) neurotransmission, the effect of histamine (0.1μM to 100 μM, Sigma) was also examined. To assess role of dopamine receptor activation on spontaneous activity, the effect of 1μM D1 agonist, A77636 ((1*R*-*cis*)-1-(aminomethyl)-3,4-dihydro-3-tricyclo[3.3.1.13,7]dec-1-yl-[1*H*]-2-benzopyran-5,6-diol hydrochloride; Tocris Bioscience, Bristol, UK) and 1 μM D2 agonist, ropinirole hydrochloride (Sigma-Aldrich, Poole, UK) were determined respectively on the amplitude and the frequency of spontaneous contraction of the isolated detrusor strips.

To assess any neurogenic alterations following MPTP treatment, detrusor strips were contracted indirectly by electrical field stimulation (EFS) delivered through a pair of platinum electrodes placed on either side of the detrusor strips in the organ bath. Detrusor strips from drug-naïve normal and MPTP-treated common marmosets were stimulated with trains of 20 pulses at 0.25, 0.5, 1.0, 2.0, 4.0, 8.0, 20 and 40 Hz and pulse duration of 0.2 ms at supramaximal voltage (50 V) once every 5 minutes. Contractile responses to EFS were evoked in the absence or in the presence of 1μM atropine (atropine sulphate, Sigma-Aldrich) to assess the contribution of the cholinergic component to the EFS-evoked contractile responses. Atropine was administered 30 min before EFS-evoked responses. The contractile responses to EFS were fully abolished by tetrodotoxin (TTX, Sigma) 1.0 μM, thus confirming the neurogenic nature of EFS-evoked contractile responses as described previously [22].

### Immunohistochemistry

Directly after perfusion and removal of bladder, the brains were removed, and placed in 4% buffered paraformaldehyde and fixed for further 48 h, washed in 0.1 M PBS, and cryoprotected in 30% sucrose solution for 4–6 days. Coronal sections from the blocks containing substantia nigra were cut at 30 μm using a Leica freezing microtome and these were kept free-floating in 0.1 M PBS containing 0.01% sodium azide until processed for immunohistochemistry.

To determine the extent of dopaminergic neural loss in substantia nigra, the sections were processed for tyrosine hydroxylase (TH) immunohistochemistry using polyclonal anti-tyrosine hydroxylase (TH) (Pel-freeze, Rogers, USA; 1:500) and avidin-biotin peroxidase complex immunohistochemistry employing rabbit ABC Vectastain kit (Vector Laboratories) as described previously [24]. Immunoreactivity was observed using 3,3-diaminobenzidine (DAB, Sigma) as the chromagen.

### Cell counting

The number of tyrosine hydroxylase-immunoreactive (TH-ir) neurones at the level of the third nerve was derived from the average of the counts of the total TH-ir neurones in three to seven adjacent sections. We have already shown that there is a close correlation between manual counting in this manner and unbiased stereology using the dissector method [25]. Based on the counts of dopaminergic neurones throughout the SN at regular 100 μm intervals, we have previously shown that the third nerve rootlets provide a reliable anatomical landmark at which the extent of cell loss can be accurately assessed and the extent of cell loss at this point is reflective of cell loss throughout the entire structure [25]. The extent of dopamine neuronal loss was estimated by counting the number of TH-ir SN neurones at the level of the third nerve rootlets on the lesioned side compared with the control side of the SN. All cells that appeared severely deformed were excluded from the counts.

### Data analysis

The data for manual TH-ir neuronal counts in the SN from each treatment group was expressed as mean ± standard error of mean (s.e.m.). The differences in SN cell counts as well as the differences in the amplitude and frequency of spontaneous contractions in tissues obtained from the naïve and MPTP animals were compared using un-paired Student’s t-test using Prism 6.0 software (GraphPad, San Diego, CA USA). For the amplitude of contractions, frequency of stimulation and the EFS-evoked contractions each value represented the mean of replicate detrusor strips running in parallel (n = 3–4) for each animal. The overall mean ± s.e.m was determined from the individual mean replicate values of all animals in their respective groups. Where multiple groups of data in concentration response curves or where the contractile responses to different stimulation frequencies were compared, a two-way ANOVA followed by Bonferoni’s comparison *post hoc* test was used. Differences were considered statistically significant at P <0.05.

## Results

### Gross morphology of bladder

**Fig 1:**
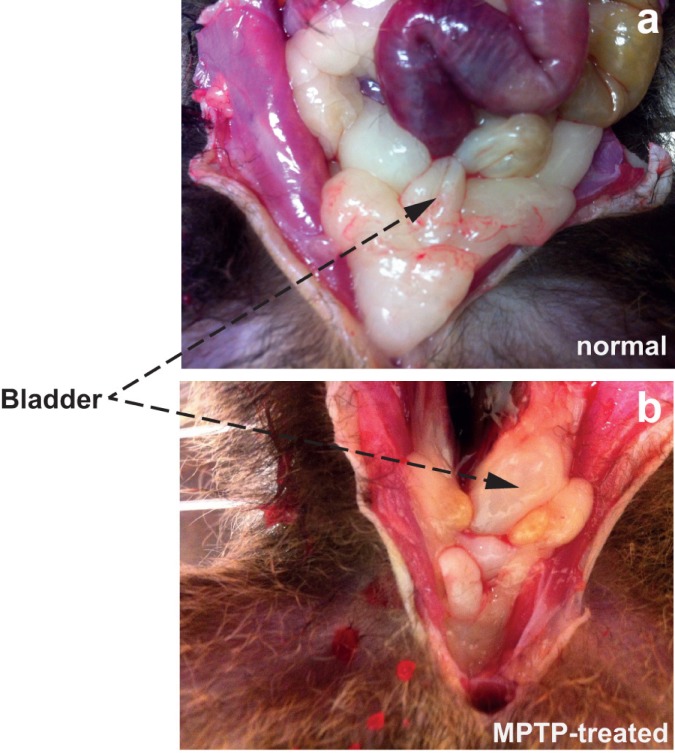
**In situ localisation of normal (a) and MPTP-treated bladder in the hypogastric region of the common marmoset.** Invariably, the bladder of the MPTP-treated common marmosets were larger in size.

Initial macroscopic observation of the urinary bladder in freshly culled animals prior to dissection showed distinct but variable bladder enlargement in MPTP-treated common marmosets compared to normal control animals (Fig 1). Examination of sections through the detrusor showed no remarkable morphological differences in the tissue obtained from the MPTP treated animals (S1 Fig).

### Nigral TH-ir

**Fig 2:**
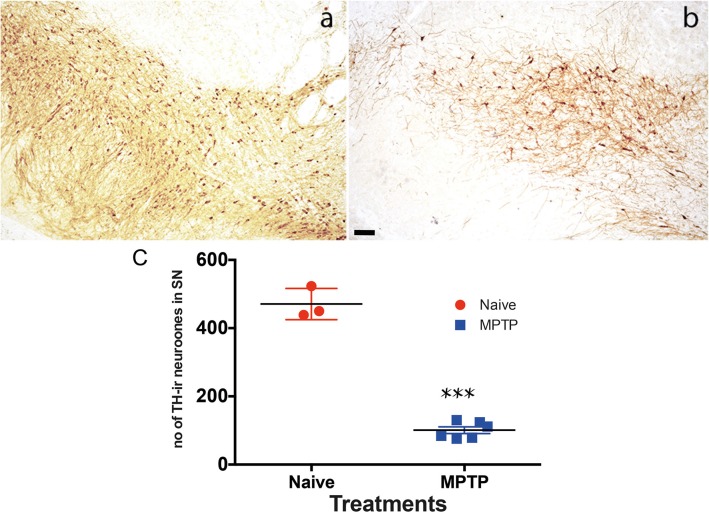
Tyrosine hydroxylase immunoreactivity in the substantia nigra (SN) of normal (a) and MPTP-treated common marmosets. TH-immunoreactive neurones in normal, drug naive (a) and MPTP-treated (b) substantia nigra were significantly reduced at the level of 3^rd^ cranial nerve following MPTP treatment. Each data point represents mean ± sem (n = 7) ***P<0.001(c). The scale bar represents 200 μm.

Analysis of the tyrosine hydroxylase immunoreactivity in the substantia nigra showed a marked reduction in staining in all MPTP-treated animals (Fig 2). Following MPTP treatment there was approximately 80% reduction in the number of TH-ir neurones in the substantia nigra (SN) (normal SN: 471 ± 26.5, n = 6; MPTP SN: 100 ± 9.7, n = 7, TH-ir).

### Effect of agonists on detrusor contraction or relaxation

**Fig 3:**
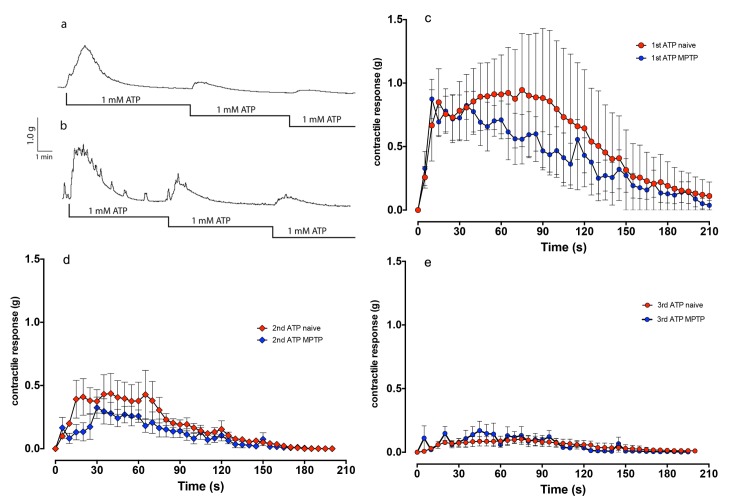
**Contractile response of the isolated detrusor strips from normal (a) and MPTP-treated common marmoset (b) to ATP.** Traces showing cumulative application of ATP produced similar contractile responses in both tissues, which were equally prone to desensitisation to subsequent ATP application. In panels c-e, the time course of the contractions to the first (c), second (d) and the third (e) application of ATP suggests that there were no significant differences between responses of the tissues obtained from normal (red data points) and MPTP-treated animals. Each data point represents mean ± sem (n = 4).

Application of 1 mM ATP led to a short-lasting contraction of the detrusor strips of the normal and MPTP-treated animals. Peak contractile response in strips from normal and MPTP-treated animals was: 1.35±0.29g, n = 4 and 1.03±0.19g, n = 4 respectively (t-test; p = 0.374). Subsequent re-application of ATP produced a progressively smaller contraction in both strips (Fig 3A and 3B). Neither the temporal pattern of contractions or the magnitude of contractions differed between the strips from normal or the MPTP-treated animals (Fig 3C–3E).

Application of noradrenaline (NA, 10μM) produced relaxation of the basal resting tone of the smooth muscle in both normal and MPTP detrusor strips. In the normal detrusor NA relaxed the detrusor (-0.42±0.05 g, n = 7) while in the MPTP detrusor this was -0.52±0.05 g, n = 7 (P = 0.146).

**Fig 4:**
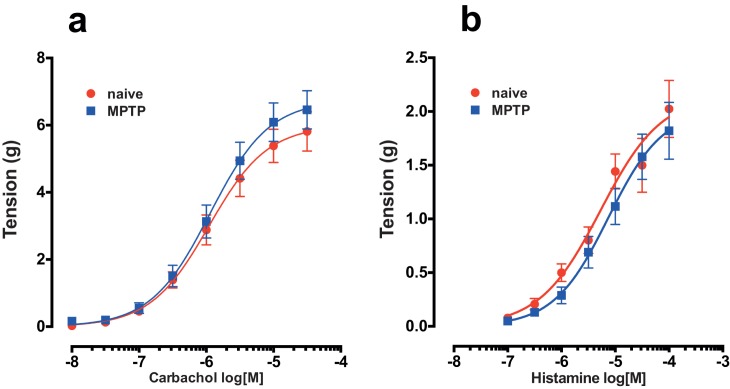
**Concentration-response effects of carbachol (a) and histamine (b) on detrusor strips from drug-naïve control and MPTP-treated animals.** Each data point represents mean ± sem (carbachol, n = 28; histamine n = 7).

There was no significant difference in the concentration-dependent contraction to carbachol in the detrusor strips of both normal and MPTP-treated animals (Fig 4A; cumulative application of carbachol in the range of 10 nM to 30 μM led to a concentration dependent contraction of the detrusor strips of both normal and MPTP-treated animals. There were no significant differences in the carbachol concentration / response curves of the normal and MPTP treated tissues (P = 0.1123; F_1-364_ = 2.53; 2-way ANOVA).

Cumulative application of histamine (100 nM to 100 μM) produced smaller contractions in comparison to carbachol (Fig 4B). However, similar to carbachol, histamine concentration response curves did not differ between normal and MPTP strips (Fig 3B, P = 0.136; F_1,238_ = 2.23; 2-way ANOVA).

### Detrusor spontaneous activity and the effect of D_1_/D_2_ receptor agonists

**Fig 5:**
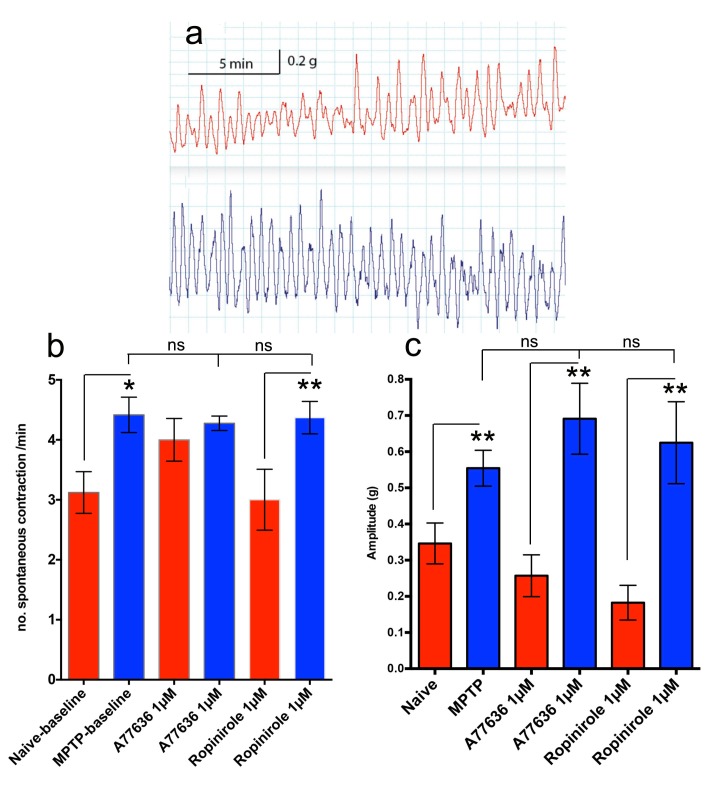
**Spontaneous contractile responses (a) in isolated drug-naïve (red trace) and MPTP-treated common marmoset detrusor strips (blue trace).** The strips from MPTP-treated animals exhibited greater frequency (b) and amplitude (c) of contraction compared to detrusor strips isolated from drug-naïve control animals or in the presence of selective D1 and D2 receptor agonists A77636 and ropinirole respectively. Each data point represents mean ± sem (n = 4–7) *p<0.05; **P<0.001; ns, not significant.

Thirty minutes after equilibration in organ baths, detrusor strips from normal and MPTP-treated marmosets exhibited spontaneous contractile responses (Fig 5A). Both the frequency and the amplitude of spontaneous contractions were modestly but significantly greater in the detrusor strips of the MPTP-treated animals (5b, c). Application of the dopamine D_1_ agonist, A77636 (1μM) or D_2_ agonist ropinirole (1μM) had no significant effect on either the tone, frequency (Fig 5B) or amplitude (Fig 5C) of contractile response of the isolated detrusor strips.

### Effect of electrical field stimulation (EFS)

**Fig 6:**
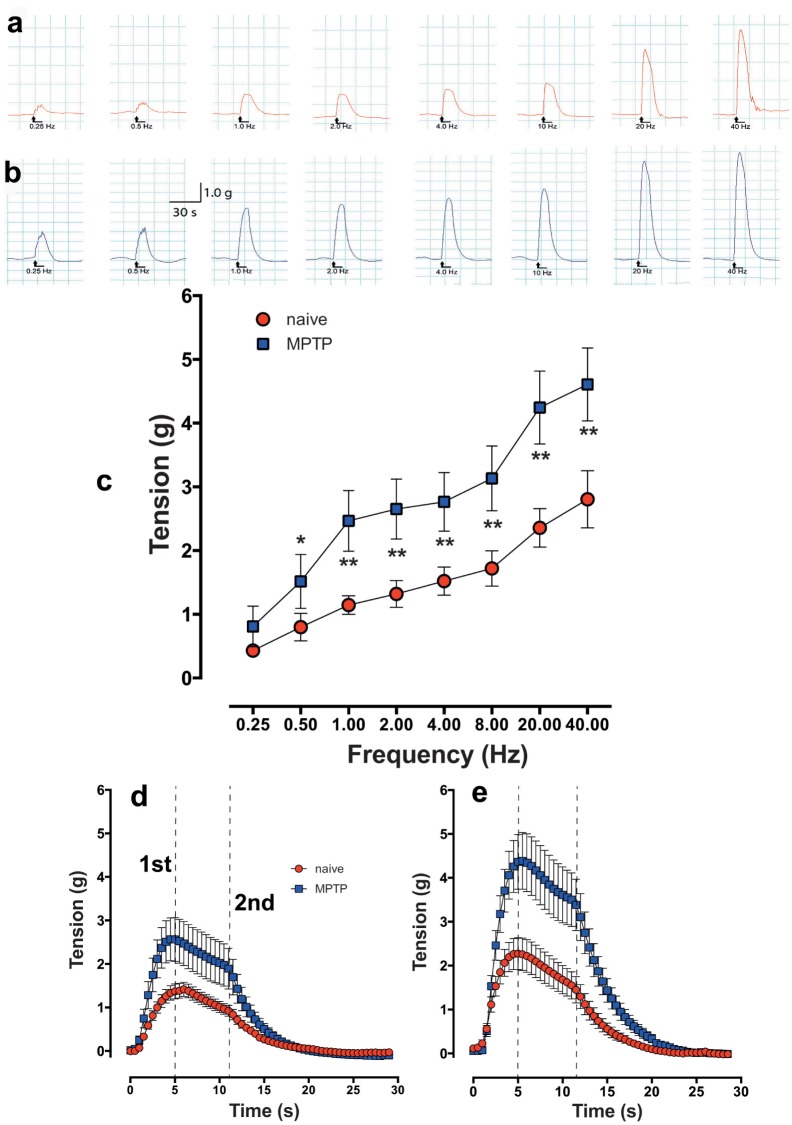
Frequency dependent increase in contractile response of isolated detrusor strips in response to EFS trains of 20 pulses at frequencies ranging from 0.25 to 40 Hz. Representative traces showing contractile responses of detrusor strips from drug-naïve (a) and MPTP-treated common marmoset (b) in response to trains of 20 pulses at frequencies ranging from 0.25 to 40 Hz. EFS-evoked contractile responses of MPTP-treated detrusor was larger at all stimulation frequencies above 1.0 Hz (c). Time course of EFS-evoked contractile responses of detrusor strips from normal and MPTP-treated common marmosets at 4 Hz (d) and 40 Hz (e) shows a biphasic contractile profile with two distinct 1^st^ and 2^nd^ peaks. The vertical lines marked 1^st^ and 2^nd^ indicate peak phasic and tonic responses respectively. Each data point represents mean ± sem (n = 19–22) * P<0.05, **P<0.005.

In response to EFS at increasing stimulation frequencies (50V, 0.2 ms pulse width and 0.25 to 40Hz trains of 20 pulses), the strips of detrusor from the normal and the MPTP-treated animals produced frequency-dependent increase in contractile tensions (Fig 6). In a few experiments, it was shown that EFS-evoked contractile responses were fully TTX sensitive (not shown). In MPTP-treated detrusor strips, contractile responses to EFS were significantly greater at all frequencies of stimulation beyond 0.25 Hz (Fig 6A–6C). When the responses at low, 4.0 Hz (20 pulses delivered for 5s) and high, 40 Hz (20 pulses delivered for 1s) frequencies were expanded (Fig 6D and 6E respectively), the contractile responses of normal and MPTP strips resulted in a similar contractile profile despite different EFS duration of stimulation and appeared biphasic: a sharp phasic contraction, peaking at 4 to 5s followed by a tonic shoulder lasting between 12 to 13s before the contractile response was extinguished completely. Regardless of the frequency of stimulation used, the timing of the appearance of the phases of the contraction remained constant, but the detrusor strips from the MPTP-treated animals exhibited markedly larger phasic peak and the latter tonic component was more clearly defined than the responses obtained from the detrusor strips of the untreated animals.

### Effects of atropine on EFS-evoked responses

**Fig 7:**
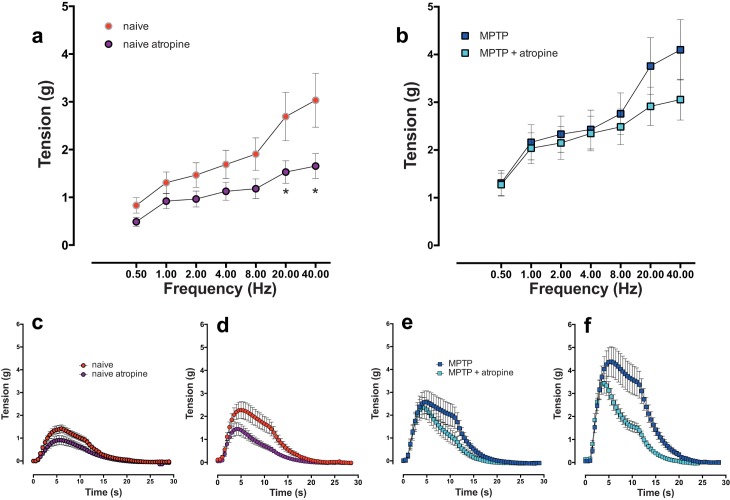
Frequency dependent increase in contractile response of isolated detrusor strips in response to trains of 20 pulses at frequencies ranging from 0.25 to 40 Hz in the absence or in the presence of 1 μM atropine in normal and equivalent tissues from MPTP animals respectively. In detrusor strips from drug naïve animals, atropine significantly inhibited peak contractile responses at frequencies above 20 Hz (a). In strips from MPTP-treated animals (b), atropine did not significantly decrease peak contractions. Time course of EFS-evoked contractile responses of detrusor strips from normal detrusor strips at 4 Hz (c) and 40 Hz (d) shows a biphasic contractile profile. In this tissue both phases of contraction were reduced by atropine while in MPTP tissues, atropine inhibited the second phase to a greater extent at the 4 Hz (e) and 40 Hz (f). Each data point represents mean ± sem (n = 5) * P<0.05.

In the detrusor strips from the normal animals, 1μM atropine reduced the peak EFS-evoked contractile responses at all frequencies (Fig 7A and 7B) but the level of reduction was statistically significant only at high stimulation frequencies (20 and 40 Hz; Fig 7A). Examination of the expanded time traces at 4 Hz and 40 Hz in strips obtained from the normal animals showed that atropine reduced both the early phasic and the later appearing tonic component to a similar extent (Fig 7C and 7D). However, in the strips from the MPTP-treated animals, the peak EFS-evoked contractions appeared less sensitive to atropine (Fig 7B). Although the mean peak contractile tension was reduced, but these were not statistically significant at any of the stimulation frequencies studied. In this group, examination of the expanded time traces at 4 Hz and 40 Hz showed that while atropine did not significantly reduce the phasic contractile component, the later appearing tonic component of the response was more markedly reduced (Fig 7E and 7F).

**Fig 8:**
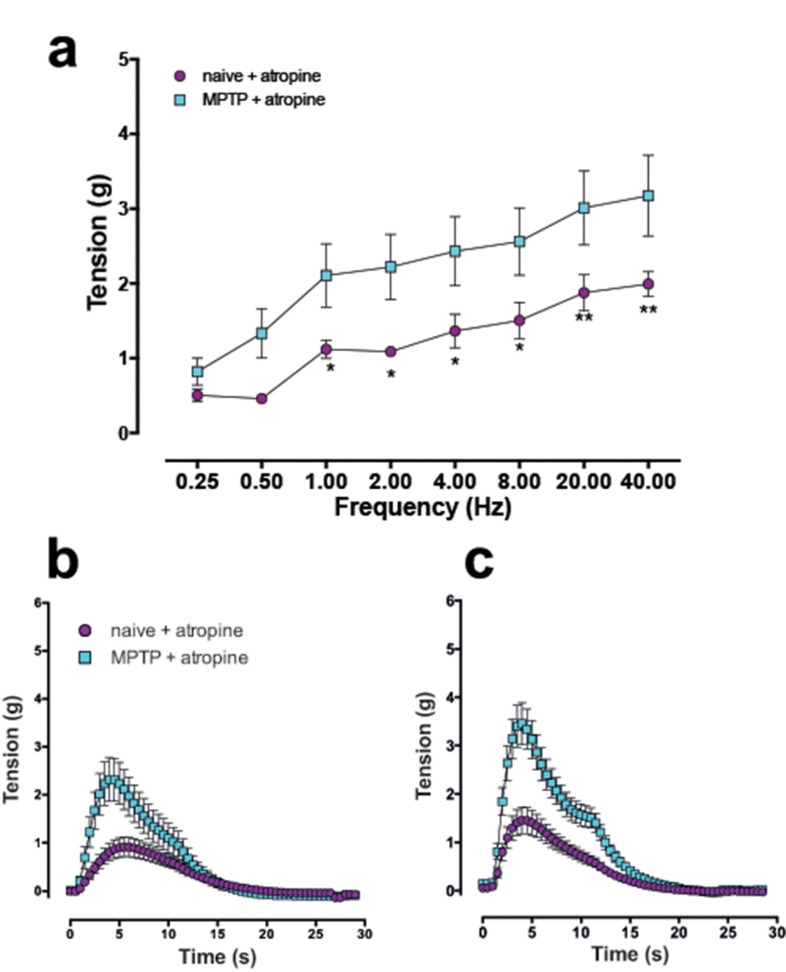
**Frequency dependent increase in contractile response of the isolated detrusor strips** in response to trains of 20 pulses at frequencies ranging from 0.25 to 40 Hz in the in the presence of 1 μM atropine in normal and the equivalent tissues obtained from MPTP-treated animals (a). Expanded time-course data shows that the first phase of contraction was markedly larger in MPTP strips compared to detrusor strips from normal drug naïve animals at 4 Hz (b) and 40 Hz (c). Each data point represents mean ± sem (n = 4–5) * P<0.05, **P<0.005.

**Fig 9:**
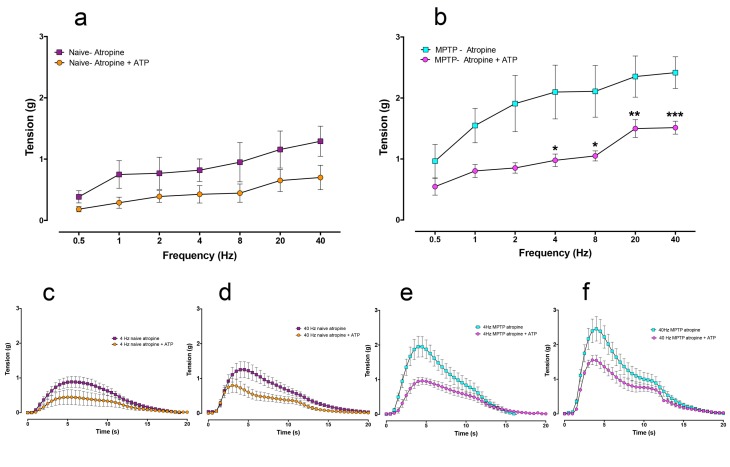
Frequency dependent increase in the atropine-resistant contractile response of the isolated detrusor strips in response to trains of 20 pulses at frequencies ranging from 0.25 to 40 Hz in the absence or following ATP-desensitisation (x3, 1mM ATP). In the detrusor strips from drug naïve animals (a, c, d), ATP-desensitisation non-significantly inhibited peak contractile responses throughout the range of frequencies used (a) whereas in the strips obtained from MPTP-treated animals (b), the contractions were significantly decreased at frequencies beyond 4Hz. Time course of EFS-evoked contractile responses of detrusor strips from normal detrusor strips at 4 Hz (c) and 40 Hz (d) shows a greater inhibition of the primary peak at 4 and 40 Hz in the tissues obtained from MPTP-treated animals (e and f) compared to tissues from normal animals (c and d). Each data point represents mean ± sem (n = 4) * P<0.05, **P<0.005, ***P<0.001.

When the atropine-resistant contractile components were compared, the responses in the detrusor strips of the MPTP-treated animals were significantly greater at all stimulation frequencies beyond 0.5 Hz (Fig 8A). When the time course of each atropine-resistant EFS-evoked contraction at 4 Hz and 40 Hz were extended, the phasic component as well as the secondary tonic phase “shoulder” was markedly larger in the MPTP strips. However, the increase in the magnitude of the secondary contractile phase (the “shoulder”) was relatively modest by comparison (Fig 8B and 8C). In another set of experiments, repeated administration of ATP to desensitise the purinergic receptors (see Fig 3) and administration of atropine (1μM) to block the cholinergic responses reduced but did not abolish the EFS-evoked contractions (Fig 9). In normal tissues, combination of ATP-mediated desensitisation and cholinergic blockade resulted in approximately 50% reduction of the EFS-evoked contractile responses (Fig 9A, 9C and 9D). However, the reduction of the primary peak responses at the various stimulation frequencies did not reach statistical significance [F (1,6) = 1.897; P = 0.221]. In the tissues obtained from the MPTP-treated animals (n = 4) however, overall the magnitude of the atropine resistant contractions, was significantly reduced (Fig 9B, 9E and 9F) following ATP desensitisation and atropine-induced blockade at 4 Hz and beyond [F (2,9) = 4.694; P = 0.0403].

## Discussion

Bladder dysfunction is a common non-motor symptom of PD characterised by bladder hyperreflexia that can be reproduced in rats and primates through destruction of the dopaminergic nigro-striatal pathway [6, 8–10]. In an *in-vivo* setting, hyperreflexia can be manipulated by dopamine agonists, but in PD, dopaminergic therapy is less effective and anticholinergics are usually employed to reduce micturition. This study is the first to investigate the possibility that some components of hyperreflexia in a relevant model of PD is precipitated at the level of bladder smooth muscle as a consequence of impaired basal ganglia function. This study was carried out in the MPTP-treated common marmosets to determine the changes in detrusor contractility and the neurotransmitter function. Inevitably, the range of investigations that could be carried out in a primate species, means that this study has uncovered previously undescribed local changes in bladder function that will need subsequent investigation in more focussed experiments. It should be emphasised that while primate research has had and continues to have a crucial role in our understanding of the pathological features of PD and the developments of novel treatments for this disorder, the ethical issues surrounding the use of primates in biomedical research, costs and the labour-intensive nature of maintenance and veterinary care places an important limitation on the range and the extent of studies that could be carried out in this species.

We have previously reported the occurrence of bladder hyperreflexia in MPTP-treated primates associated with increased micturition frequency [6] and in this study, one of the first observations following necropsy was the increased size of bladders in MPTP-treated animals. Although we did not quantify changes in the morphology of the bladder, but changes in the appearance of the bladder has been reported in various models of PD elsewhere [26, 27]. In the current experiments where MPTP treatment resulted in >80% loss of dopaminergic neurones in the substantia nigra, loss of nigrostriatal dopaminergic innervation appears to lead to long-term changes in bladder anatomy and neural physiology. The contractile response of isolated detrusor muscle, which is devoid of extrinsic innervation to smooth muscle, showed an increased frequency and amplitude of spontaneous contractile activity in tissues from MPTP-treated animals. The changes in contractility did not appear to be due to altered responsiveness to histamine or carbachol suggesting that histaminergic and cholinergic receptor response remains unaltered. Furthermore, the relaxing effect of noradrenaline was also unaltered in the bladder tissue obtained from MPTP-treated animals. When ATP was applied, the contractile responses between normal and muscle strips from MPTP-treated animals did not differ. Previously, application of ATP to marmoset detrusor strips was shown to produce a biphasic contractile response: an initial contraction followed by relaxation through proposed classical P2X and P2Y receptors respectively [28]. However, we did not observe this phenomenon in tissues from either normal or MPTP-treated marmosets. Therefore, taking these observations together, it appears that MPTP treatment had no effect on the muscarinic or purinergic receptor density, binding characteristics or distribution and signal transduction pathways. In addition, the selective D_1_ and D_2_ receptor agonists A77636 and ropinirole respectively, had no significant effect on the frequency or the amplitude of spontaneous detrusor activity. This suggests that there is no local dopaminergic contribution to detrusor activity in contrast to the centrally mediated effects on hyperreflexia observed in the whole animal. However, in contrast to the present finding, it was shown recently that following lesioning of the nigrostriatal tract with 6-hydroxydopamine, rat detrusor strips produced greater contraction to both methacholine and ATP [26]. The apparent difference between our data in common marmosets and those in the rat [26] require further investigation but may be related to differences in the species or the differences in experimental paradigms. The observed differences cannot be attributed to muscle hypertrophy as care was taken to prepare strips of similar size and proportions and the agonist induced contractile responses were not different in the same tissues that responded differently to EFS. Moreover, gross examination of paraformaldehyde fixed sections of the detrusor strips used in the current experiment showed no remarkable differences in muscular arrangement and organisation (S1 Fig).

EFS in the detrusor strips produced robust frequency dependent contractions that were fully TTX sensitive, thus these responses were deemed to be of neurogenic origin. The tissues obtained from normal and MPTP-treated marmosets responded differently to neurogenically mediated contractions. In agreement with the recent study in the rat [26], the strips from MPTP-treated animals exhibited significantly greater peak contractions in response to EFS at all stimulation frequencies above 0.5Hz. Since the contractile innervation of the detrusor is both cholinergic and non-cholinergic (presumed to be purinergic), we investigated whether the changes were due to increased contribution of either or both components. When the cholinergic component was blocked using atropine in strips obtained from normal animals, there was a significant reduction of peak contractions but this reduction was not observed in strips from MPTP treated animals. These contractile responses appeared to be bi-phasic. The first phase was predominantly non-cholinergic as atropine only had a small effect while the second phase was predominantly cholinergic as atropine tended blocked it to a greater extent. These findings suggest that the increased contractility seen following MPTP treatment was dependent at least partially on the enhancement of non-cholinergic/purinergic transmission. Furthermore, since there were no differences in the effects of carbachol or ATP, then the observed increase of the contractile response to EFS must be due to presynaptic mechanisms rather than postsynaptic effects. This would fit with evidence that in neurogenic bladder and hypertrophic unstable bladder syndromes, TTX-sensitive but atropine resistant, purinergic contractions are increased [16, 29–31]. In neurogenic bladder, it was shown that there was a marked increase in the purinergic contribution to parasympathetic control of bladder contractility [15]. Moreover, the observation in the current study that following atropine blockade, contractile responses in detrusor strips from MPTP-treated animals were significantly larger than in detrusor muscle from normal animals suggests a dynamic interplay between the cholinergic and purinergic neurotransmitters in this tissue. Indeed, following MPTP treatment, atropine failed to reduce peak response significantly, although there was a marked reduction of the later occurring secondary peak (see Fig 6I and 6J). This suggests that the cholinergic contribution of the first phase was reduced but the non-cholinergic, purinergic contribution increased. Importantly, when the atropine treated tissues were desensitised to multiple prolonged ATP exposures, a non-cholinergic, non-purinergic contractile component remained in the strips from all animals, finding that mirrored earlier studies in the rat [32]. However, the difference between the primary contractile peak in the atropine resistant component and the atropine and ATP resistant component was greater in the detrusor strips from MPTP-treated animals (Fig 9). This suggests that there was a greater level of purinergic component in the tissues obtained from the MPTP-treated animals, which is in line with the observations in neurogenic bladder [33].

These initial experiments need to be looked at in the light of how such changes in the contractility and neuronal responsiveness of isolated detrusor might occur. The obvious explanation is that they are secondary response to the loss of basal control of bladder function. The MPTP induced loss of dopaminergic tone might dysregulate pontine and dorsal vagal output centrally, which might in turn adversely affect the local inhibitory tone to the smooth muscle [34]. It cannot be ruled out that MPTP (or MPP+) has some peripheral effect on catecholaminergic innervation of the bladder or destroys dopaminergic regulation of bladder function at the level of the spinal cord, although none of these possibilities have ever been reported. The changes observed might be secondary to the effects of loss of basal ganglia function in the complex control of bladder function and represent adaptive changes to that loss. Since these are the first experiments to be undertaken on isolated bladder muscle from MPTP-treated primates, only a limited analysis of pre- and post-synaptic transmission was possible and other neurotransmitter/neuromodulatory systems need to be investigated. For example, detrusor muscle contractility is also regulated by the inhibitory tone from nitric oxide (NO) [34–36] and NO synthase inhibition by L-NAME enhances the amplitude of detrusor contractility [36]. Consequently, it is conceivable that in the detrusor of the normal animals, EFS-evoked contraction is the sum of the effects of the excitatory as well as the inhibitory neurotransmitters. One other caveat that must be introduced is the limited pathology produced by MPTP treatment compared to the widespread cell loss in PD that might also contribute to bladder dysfunction. PD is considered to be a synucleinopathy and α-synuclein positive inclusions have been found in the bladder of the α-synuclein overexpressing mice [27]. Since these mice show bladder distension, it might be that the alterations in bladder function that occur in PD are part of the peripheral pathology of PD rather than being centrally mediated.

In conclusion, the results of this study shows that compared to the isolated detrusor muscle preparations from normal common marmosets those obtained from MPTP-treated animals with marked striatal dopaminergic neurone loss show enhanced spontaneous contractile activity and contractility in response to EFS. Since exogenously applied contractile agonists (carbachol, ATP, histamine) and noradrenaline did not distinguish between normal and MPTP detrusor, it is suggested that MPTP-treatment did not affect receptor population, signal transduction pathway or the physiology of the smooth muscle receptor/effector coupling. The observed increase in detrusor contractility in response to EFS appears to be due in part to enhancement of the purinergic/non-cholinergic transmission, loss of inhibitory transmitter or the dysfunction of the inhibitory signal transduction or a combination of these. Although the mechanisms of spontaneous rhythmic activity and nerve-evoked contractions differ but the fact that following MPTP treatment both increase in magnitude suggest that these changes might be grounded in a common signalling molecule such as ATP or alterations in smooth muscle Ca^2+^ [37].

It is important to note that the observations in the human bladder suggest that the contribution of the cholinergic transmission to the contractile response of the detrusor muscle is much greater than the purinergic/non-cholinergic transmission [19]. Indeed, the relative contribution of the cholinergic and non-cholinergic transmission in the normal marmoset tissues seems to be more closely aligned to that seen in the rat [18, 22], which suggests that it would be equally valid to compare the contractility of normal and parkinsonian detrusor also in the rodents. However, it is important to note that differences in gene expression profile between different species, even in those as close as rats and mice would likely to impact the physiological parameters and functional responses. Consequently, any physiological, anatomical and functional differences between the detrusor strips obtained from 6-OHDA treated rats [26] and those from MPTP-treated common marmoset in the present study is not surprising. Moreover, unlike primates, rats generally do not exhibit most of the cardinal symptoms of PD, the brain of rodents are anatomically different to primates (e.g. no differentiation of the caudate nucleus and putamen; no subdivisions of the globus pallidus intra and externa to name a few) and neurotoxins that produce nigrostriatal lesions are given unilaterally i.e. into one hemisphere thus producing only hemiparkinsonism–so not a bilateral Parkinson’s disease per se, so direct comparisons between rats and primates should be carried out cautiously.

The results of this study suggest that as well as the documented central changes that take place in PD, local changes at the level of the presynaptic neuro-effector junction muscle might make a significant contribution to hyperreflexia and bladder instability that is frequently observed in PD. Moreover, targeting the non-cholinergic as well as or instead of the cholinergic neurotransmission might provide a better treatment for bladder hyperreflexia seen in PD.

## Supporting information

S1 Fig Gross morphology of detrusor strips from normal and MPTP-treated animals. Representative examples of 10 μm transverse sections of paraformaldehyde fixed, paraffin-embedded detrusor from normal and MPTP-treated common marmosets. There were no remarkable alterations in the tissue morphology of the detrusor muscle obtained from the MPTP-treated animals compared to those from normal animals.
(TIF)

## Abbreviations

ATP: adenosine triphosphate
A_2a_: adenosine A2 receptor
EFS: electrical field stimulation
MPTP: 1-methyl-4-phenyl-1,2,3,6-tetrahydropyridine
NA: noradrenaline
NO: nitric oxide
NANC: non-adrenergic non-cholinergic
PD: Parkinson’s disease
SN: substantia nigra
TTX: tetrodotoxin
