# Supplementary figures and images for: Altered detrusor contractility in MPTP-treated common marmosets with bladder hyperreflexia
Source: PLoS One. 2017 May 17;12(5):e0175797. doi: 10.1371/journal.pone.0175797 (PMC5435136; doi:10.1371/journal.pone.0175797)

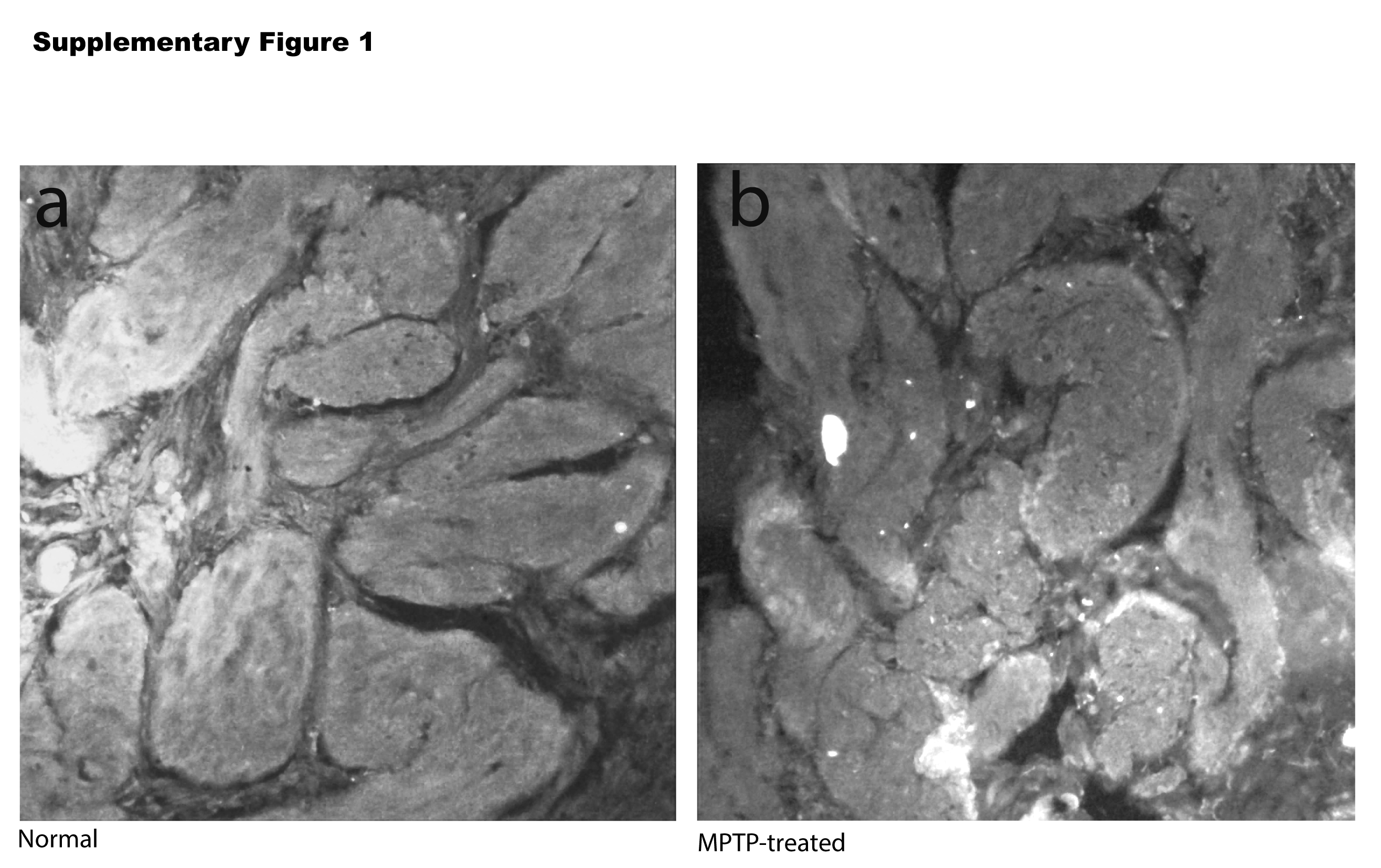

Supplement: S1 Fig — Representative examples of 10 μm transverse sections of paraformaldehyde fixed, paraffin-embedded detrusor from normal and MPTP-treated common marmosets. There were no remarkable alterations in the tissue morphology of the detrusor muscle obtained from the MPTP-treated animals compared to those from normal animals. (TIF) [file pone.0175797.s001.tif]
